# Multilevel Factors Influencing Perceived Barriers to Adjuvant Endocrine Therapy Among Breast Cancer Patients at Medication Onset

**DOI:** 10.3390/ijerph22050734

**Published:** 2025-05-06

**Authors:** Timothy Cocozza, Rita Smith, Ana Maria Lopez, Shari Rudoler, Rachel Slamon, Tingting Zhan, Jazmarie L. Vega, Minal Dhamankar, Aruna Padmanabhan, Suzanne M. Miller, Kuang-Yi Wen

**Affiliations:** 1 Sidney Kimmel Medical College, Thomas Jefferson University, Philadelphia, PA 19107, USA; 2 Department of Medical Oncology, Thomas Jefferson University, Philadelphia, PA 19107, USA; 3 Department of Radiation Oncology, Thomas Jefferson Torresdale Hospital, Philadelphia, PA 19107, USA; 4 Department of Pharmacology, Physiology, and Cancer Biology, Thomas Jefferson University, Philadelphia, PA 19107, USA; 5 Fox Chase Cancer Center, Temple University Hospital, Philadelphia, PA 19111, USA; 6 Division of Medical Oncology, Einstein Medical Center, Philadelphia, PA 19107, USA

**Keywords:** breast cancer, endocrine therapy, adherence barriers

## Abstract

**Purpose:** Adjuvant endocrine therapy (AET) significantly diminishes recurrence and mortality risks in hormone receptor-positive breast cancer (BCa) patients. Nonetheless, suboptimal adherence and premature discontinuation during the initial year of treatment undermine these positive outcomes. This study aims to understand the potential diverse factors associated with perceived barriers to AET compliance at the onset of medication. **Methods:** We assessed perceived barriers to AET using the ASK-20 instrument for BCa patients initiating AET within 3 months. Our survey also included demographic variables (e.g., musculoskeletal symptoms) and clinical traits (e.g., medication type). Stepwise regression analyses were employed to elucidate the links between multilevel factors and perceived barriers to AET adherence. **Results:** In our cohort of 272 women, the mean ASK-12 score was 38.2 +/− 9.2 (range 20–100). In the multivariable regression model, greater perceived barriers to adhering to AET were found to be associated with African American ethnicity (Β  =  2.47; 0.53–4.21; *p*  <  0.05), lower self-efficacy in medication management (Β  =  −0.80; −1.03–−0.58; *p*  <  0.001), higher psychological distress (Β  =  2.79; 0.61–4.97; *p*  <  0.05), increased reported distress related to musculoskeletal side effects (Β  =  0.64; 0.31–0.97; *p*  <  0.001), weight gain symptoms (Β  =  0.61; 0.18–1.03; *p*  <  0.05), less family support (Β  =  −0.38; −0.53–−0.13; *p*  <  0.05), and higher levels of concern pertaining to AET (Β  =  0.64; 0.41–0.87; *p*  <  0.001). **Conclusions:** Modifiable factors are associated with women’s perceived barriers to AET at the onset of treatment. Proactively addressing patient concerns about AET, improving self-regulatory skills for medication management and family support, and enhancing symptom management strategies, along with addressing distress at the onset of treatment, hold promise for mitigating barriers to AET. Furthermore, recognizing the distinctive challenges faced by African American subgroups is crucial, necessitating culturally tailored interventions to reduce potential disparities and ensure equitable access and adherence to AET. Continued research and tailored interventions are important for optimizing outcomes and reducing the impact of modifiable barriers on AET adherence.

## 1. Introduction

Breast cancer (BCa) remains the most frequently diagnosed cancer worldwide [1]. Endocrine therapies have improved prognosis for approximately 70% of women with estrogen and progesterone receptor-positive tumors [2]. Five years of endocrine therapy was previously standard; however, recent guidelines recommend extending endocrine therapy to at least 10 years for certain patients, posing greater adherence challenges [3]. Data show that non-adherence rates range from 28% to 59%, with inconsistency or discontinuation rates being particularly high during the first critical year after initial prescription [4]. Non-adherence to AET increases the risk of mortality and recurrence as well as the risk of a significant loss in quality-adjusted life-years [5,6]. Given that adherence to the full course of AET (≥5 years) plays an 2.0important role in reducing BCa recurrence risk by 40% and mortality by 31% [7,8], and since early adherence is a strong predictor of long-term persistence [9,10], it is essential to develop strategies to promote compliance from the initiation of treatment [11].

Demographic and clinical characteristics may be key in identifying subgroups at risk for non-adherence at the onset of treatment, guiding the development of tailored interventions during early survivorship. For example, research has suggested that younger age and minority status are associated with high rates of non-adherence to AET [12]. Additionally, receipt of chemotherapy was associated with lower adherence [6]. Further, many patients do not follow their AET regimens because they cannot manage the side effects [13]. Qualitatively, a spectrum of symptoms, such as cognitive problems and hot flashes, were reported as barriers to adherence to AET [14]. 

Many patients begin AET immediately following chemotherapy or radiation, a period often referred to as the “re-entry” phase [15]. During this phase, BCa survivors frequently experience distress related to a deviation from their former personal and professional responsibilities, reduced interpersonal support, and the long-term effects of diagnosis and treatment on their physical and mental health [15]. These challenges can exacerbate the burden of starting AET, especially when chemotherapy-related toxicities have become baseline symptoms, further reducing the likelihood of early AET adherence. Additionally, the psychological impact of estrogen deprivation, a side effect of AET, can impair cognitive and emotional well-being, compounding the difficulties of continuing treatment. Further, a person’s beliefs about their medication, such as perceptions of its necessity and concerns about side effects, significantly influence adherence behaviors [16]. Research has also shown that a higher level of self-efficacy and social support are positively associated with better AET adherence [17,18], suggesting that addressing these modifiable factors may enhance adherence outcomes.

With AET guidelines recommending prolonged treatment durations, it is essential to understand the risk factors associated with perceived barriers to adherence with the ultimate goal of developing interventions to support patients during this re-entry phase. The diverse nature of barriers to AET adherence highlights the importance of patient-tailored interventions to increase adherence. To better understand the drivers of AET adherence and identify modifiable factors for intervention, the aim of this study is to explore the demographic, clinical, and psychosocial factors associated with patients’ perceived barriers to AET adherence among BCa survivors during the early re-entry phase in survivorship. Our analyses use perceived barriers at the time of medication initiation as outcomes, with demographic, clinical, and modifiable psychosocial factors serving as key predictors. By addressing these barriers during the critical re-entry period, when survivors face physical, emotional, and social challenges, interventions can be more effectively tailored to improve initial adherence and potentially sustain long-term outcomes.

## 2. Methods

### 2.1. Study Design

These analyses included data from an ongoing RCT study that aimed to understand ways to help women adhere to AET during early survivorship. All analyses used a cross-sectional design from the baseline data of the RCT study. Women completed the baseline survey either online via REDCap or via a mailed survey copy to collect demographic and clinical characteristics and psychosocial factors. The surveys took approximately 30 min to complete. Participants were compensated with a USD 30 clinic card. Additional medical characteristics were extracted from electronic medical records. This study was approved by the Thomas Jefferson University’s Institutional Review Board (IRB) and the Sidney Kimmel Comprehensive Cancer Center’s (SKCCC) regulatory committees.

### 2.2. Subjects and Setting

The inclusion criteria consisted of women ≥ 18 years of age diagnosed with stage 0, I, II, or III hormone receptor-positive BCa who had completed local definitive treatment and were within 3 months of beginning the first AET regimen. Exclusion criteria: patients were excluded if they had documented cognitive impairment that could interfere with survey completion or reliable self-reporting. Participants were recruited from the SKCCC at Thomas Jefferson University and the Fox Chase Cancer Center (FCCC) at Temple Health in Philadelphia, Pennsylvania, USA, as well as through the Cancer Support Community. The decision to focus on early medication initiation was based on evidence showing its positive association with long-term discontinuation [9,10].

**Measures**. Constructs were assessed using previously validated scales, as detailed below.

#### 2.2.1. Demographic and Clinical Characteristics

The sociodemographic variables measured in this study included age, race, ethnicity, education, marital status, household income, and work status. The clinical characteristics collected were the type of AET medication, specifically either an aromatase inhibitor (exemestane, anastrozole, or letrozole) or tamoxifen, cancer stage, whether the patient received chemotherapy or radiation therapy, and type of surgery (e.g., lumpectomy, mastectomy).

#### 2.2.2. Perceived Barriers to Adherence

The Adherence Starts with Knowledge 20 (ASK-20) questionnaire was used to identify barriers to treatment adherence [19]. The ASK-20 includes 20 clinically relevant items that assess various factors influencing medication adherence. The total ASK-20 score was derived by summing the raw item scores, with higher scores indicating greater perceived barriers to AET adherence.

#### 2.2.3. Symptom Distress

The Breast Cancer Prevention Trial (BCPT) Symptom Scale is a 42-item assessment that evaluates distress related to physical and psychological symptoms. It encompasses eight symptom domains, including hot flashes, nausea, bladder control issues, vaginal concerns, musculoskeletal pain, cognitive difficulties, weight changes, and arm-related problems [20]. These scales have been extensively utilized, including in studies on psychosocial interventions for breast cancer. Higher BCPT scores indicate higher symptom distress.

#### 2.2.4. Self-Efficacy

The Medication Understanding and Use Self-Efficacy (MUSE) survey is an eight-item questionnaire used to measure patients’ confidence in their ability to understand, manage, and correctly take their prescribed medications [21]. Self-efficacy, in this context, refers to a person’s belief in their capacity to perform medication-related tasks successfully.

#### 2.2.5. Medication Belief

The patient’s beliefs about their AET medication was measured by the Beliefs about Medicines Questionnaire (BMQ). Each subscale of the BMQ assesses a patient’s beliefs about a specific medication they are prescribed for an illness. The first scale relates to the theme Specific-Necessity (five items) [22]. For this paper, this scale measured patient beliefs about the necessity of their AET medication. The second subscale evaluates the theme Specific-Concerns (five items) to measure patient concerns about the negative effects of their AET treatment [22]. 

#### 2.2.6. Affect

The Revised Impact of Events Scale (IES-R), a validated questionnaire that evaluates subjective distress caused by traumatic events, measured cancer-related distress among participants [23].

#### 2.2.7. Social Support

The Multidimensional Scale of Perceived Social Support (MSPSS) questionnaire assessed perceptions of support received from family, friends, and significant others among cancer patients as three subscales.

### 2.3. Data Analyses

All analyses conducted utilized baseline data from an ongoing RCT. Descriptive statistics were used to summarize demographic, clinical, and psychosocial characteristics. Continuous variables were reported using means and standard deviations (SDs), while categorical variables were summarized with frequencies and percentages. To assess associations between perceived barriers to AET adherence (dependent variable: ASK 20) and demographic, clinical, and psychosocial factors (independent variables), multivariable linear regression analyses were conducted. A stepwise selection method was employed in the regression models. All statistical tests were two-sided with a significance level of α = 0.05, and *p*-values were obtained through F tests. Analyses were performed using R version 2023.

### 2.4. Results

Among the 272 women analyzed, the majority were over the age of 50 (72.4%) and identified as white (75.0%). More than half (59.9%) had some level of college education, and 65.1% were married. Regarding treatment, 77.2% of participants were prescribed an aromatase inhibitor. Cancer staging revealed that 84.7% had either stage 0 or I breast cancer. A significant proportion (74%) had previously undergone radiation therapy, while 67.2% had not received chemotherapy. Surgical history indicated that 78.6% had undergone a lumpectomy, whereas 26.3% had had mastectomy procedures. Socioeconomic factors showed that 58.8% of women reported an annual household income exceeding USD 75,000, and 51.5% were employed, either part-time or full-time (Table 1).

Participants’ ASK Adherence Barriers score ranged from 20 to 100, with a mean score of 38.2 (±9.2), indicating moderate barriers to adherence. The BMQ necessity score ranged from 5 to 20, with a mean of 15.8 (±3.4), suggesting that participants generally perceived their medication as necessary. The BMQ concern score ranged from 5 to 20, with a mean of 13.3 (±3.8), reflecting moderate concern about medications. For trauma-related stress, participants had a mean score of 20.7 (±14.8) on the IES-R, indicating a moderate level of distress. In terms of symptom burden, the BCPT subscales revealed low-to-moderate symptom severity. The vasomotor symptoms subscale had a mean of 2.7 (±2.4) in a range of 2 to 10, while the gastrointestinal symptoms subscale showed low symptom levels with a mean of 0.6 (±1.0). The bladder control symptoms subscale averaged 1.3 (±1.8), and the dyspareunia subscale averaged 1.9 (±2.3), both in a range 2 to 10. Musculoskeletal pain had a mean score of 4.7 (±3.4) out of 15, indicating moderate pain. The weight concerns and cognitive symptoms subscales had means of 2.6 (±2.1) and 3.4 (±2.9), respectively, showing relatively mild concerns. The MUSE scale, ranging from 8 to 32, had a mean score of 26.6 (±3.8), suggesting a high level of perceived self-efficacy in managing one’s medication. Lastly, the MSPSS subscales showed moderately high perceived support.

In the multivariable regression model, greater perceived barriers to adhering to AET were found to be associated with African American ethnicity (*Β*  =  2.47; 0.53–4.21; *p*  <  0.05), lower self-efficacy in medication management (*Β*  =  −0.80; −1.03–−0.58; *p*  <  0.001), higher affect (*Β*  =  2.79; 0.61–4.97; *p*  <  0.05), increased reported distress with musculoskeletal pain symptoms (*Β* =  0.64; 0.31–0.97; *p*  <  0.001) and weight gain symptoms (*Β*  =  0.61; 0.18–1.03; *p*  <  0.05), less family support (*Β*  =  −0.38; −0.53–−0.13; *p*  <  0.05), and a higher level of concern pertaining to AET (*Β*  =  0.64; 0.41–0.87; *p*  <  0.001) (Table 2).

## 3. Discussion

As far as we are aware, this study is one of the first to identify multilevel factors associated with perceived adherence barriers to AET at the start of the medication regimen. A key strength of our study is that all participants were recruited within three months of their AET prescription, allowing us to capture these barriers at the critical early stages of treatment initiation. The added burden of managing a new treatment and pre-existing side effects during this re-entry phase may become overwhelming, making it essential to understand the factors influencing perceived barriers to adherence. Overall, our participants reported moderate levels of barriers to medication at the onset of AET treatment. However, we found that greater perceived barriers were more prevalent among African American women, those with less self-efficacy and family support, individuals with higher distress and concern with medication, and those reporting more distress related to musculoskeletal pain and weight gain. These findings underscore the crucial nature of early intervention and support, particularly for patients facing increased physical and psychosocial challenges during the initial phase of AET.

The factors associated with perceived barriers to AET identified here are consistently documented in the existing AET literature. In particular, racial differences in AET perception and adherence have been observed, suggesting that African American women are more likely to report more barriers and be non-adherent than their White counterparts [24,25]. The decreased use of AET among African American women has been attributed to higher mortality levels among this group [26]. Therefore, this emphasizes the importance of further understanding relating to African American women’s adherence to AET. Studies have also shown that both biological and social factors may impact morbidity and mortality outcomes among African American women who undergo treatment for BCa [27]. Therefore, culturally tailored interventions might be important to address these disparities and perceived barriers, increasing the number of African American women who properly adhere to their AET treatment.

Symptom management is a key aspect of healthcare providers’ management of their patients’ AET regimen. However, little is known on how this aspect of care impacts AET adherence or perceptions [28]. Racial disparities also exist in reported side effects associated with AET, with African American women reporting higher rates of these side effects, which may place an additional burden on this population [29]. Our data specifically show that self-reported problems in the musculoskeletal pain and weight gain domains are associated with perceived barriers to AET adherence. Early lifestyle modifications and resources provided by oncologists, such as physical activity and dietary support, may help alleviate concerns over weight gain. Musculoskeletal pain has been identified as one of the major reasons women discontinue AET [14], particularly with an AI. Arthralgia, or joint pain, often occurs as early as two months into AI treatment and is among the most troublesome side effects, with 77% of our sample comprising women taking an AI. Thus, early interventions to mitigate AI-induced arthralgia may improve treatment adherence. Studies have reported that exercise [30] or integrative approaches [31] show initial promise in reducing arthralgia; however, these strategies will need to be further evaluated to understand whether they directly improve AET adherence.

Our study also found that participants with higher levels of emotional distress were associated with greater perceived barriers to adherence. This is particularly important to address in BCa patients initiating AET, as studies have shown that emotional distress is highest during the first two years of treatment [32], so early intervention might help prevent the escalation of distress over time, negatively impacting adherence. Emotional distress may be linked to a lack of proper coping mechanisms developed throughout the course of treatment [33], including prior therapies such as chemotherapy and/or radiation before AET initiation. Studies have shown that psychological interventions that foster positive coping mechanisms may help alleviate this risk factor and reduce adherence barriers [34].

Our study shows that BCa survivors with less perceived individual control over their medical treatment were more likely to report increased barriers to adherence. These findings are consistent with past studies linking medication adherence and self-efficacy for AET treatment [35]. Self-efficacy levels among patients represent a potentially modifiable area that can be addressed through behavioral interventions that target self-regulation and coping skills [36]. Past research has found that social support offered by family and friends promotes continued adherence to AET [37], which is consistent with our findings. Our data show that women’s health beliefs and attitudes toward AET significantly influence their perceived barriers to adherence. Negative attitudes and greater concerns about AET are associated with lower adherence in emerging studies [35]. Oncologists play a key role in addressing these beliefs by providing clear information and scheduling follow-ups to explain side effects or address concerns. Adequate information about AET’s importance, benefits, and risks is crucial for BCa survivors to understand before initiating treatment. Patient-centered communication that is culturally competent and respects patients’ backgrounds is essential for adherence. Developing tailored communication tools to enhance conversations between patients and oncologists about AET and related decisions should be a focus of future research to address patient’s concerns and beliefs.

This study has several strengths, including a diverse sample with representation from both African American and Caucasian patients. Additionally, the collection of various psychosocial factors and the focus on the initiation phase of medication provide valuable insights. However, it is plausible that adherence barriers may evolve over the course of extended therapy, particularly given the physical and psychological burdens associated with long-term use. Therefore, results may differ in a population with longer AET exposure, warranting further longitudinal research to capture changes in adherence and associated non-adherence risk factors over the full course of therapy. Further, most participants were diagnosed with stage 0 or I breast cancer in our study, which limits our ability to draw definitive conclusions about potential differences in adherence barriers by disease stage. Although the study was conducted across two cancer centers, both sites are located within the greater Philadelphia region, which may limit the generalizability of the findings to other geographic or healthcare settings. In general, the psychosocial scores were moderate rather than severe. Alternative measures of adherence were not included in this study; however, our ongoing research will explore multiple dimensions of adherence. It is important to acknowledge that the cross-sectional design of this study restricts the ability to evaluate changes over time.

Addressing adherence to AET is crucial for reducing negative health outcomes among breast cancer survivors. To comprehensively tackle AET non-adherence, understanding adherence behaviors at various time points during treatment is vital. Early intervention at the onset of medication is particularly crucial, as it can impact long-term compliance and outcomes. Recognition of early barriers to adherence may allow medical providers to address these restraints and prevent the future cessation of AET. When developing interventions to address AET barriers and non-adherence, it is essential not to adopt a one-size-fits-all approach. Instead, the unique barriers faced by diverse patient populations must be considered. This study underscores the need for multi-faceted interventions, particularly those addressing equity in AET use. To address the barriers reported in this study, several potential strategies can be implemented within clinical settings. For example, the integration of brief self-efficacy screening tools into routine care might help to identify patients at risk of non-adherence early on in treatment. Providing culturally tailored educational materials and implementing timely psychosocial support interventions may also help patients navigate concerns and reduce distress related to AET. These interventions should facilitate strategies for medication adherence while cultivating skills and enhancing self-efficacy to overcome barriers. Additionally, enhancing symptom management protocols and guidelines may reduce perceived barriers and improve symptom management. Institutional initiatives such as multidisciplinary care teams and patient navigation programs can further support sustained adherence by addressing individual- and system-level challenges across the continuum of care. Particularly for populations like African Americans or those with lower social support and higher symptom burden, multi-component interventions need to be further evaluated.

## 4. Conclusions

Non-adherence to AET has substantial implications in the long-term morbidly and mortality outcomes of BCA survivors. This study brings attention to critical risk factors acting as barriers to AET adherence. Moving forward, it will be imperative to tackle these multilevel barriers discussed in this paper and develop interventions that address the unique experiences of BCa survivors to improve AET adherence. Additionally, leveraging these insights is crucial in light of recent expanded clinical guidelines recommending a 10-year course of AET. Addressing adherence challenges early and throughout the extended treatment period is essential for improving overall outcomes and reducing the potential negative impact of non-adherence.

## Figures and Tables

**Table 1:** Participants’ demographic, clinical, and psychosocial characteristics (*N*  =  272).

*n* (%) or *n* (Mean ± SD)	
Age	
>50 years	197 (72.4)
≤50 years	75 (27.6)
Race	
African American	68 (25.0)
Caucasian	204 (75.0)
Education	
Less than college	109 (40.1)
College or above	163 (59.9)
Marital status	
Married	177 (65.1)
Single/divorced/windowed	95 (34.9)
Household annual income	
≤75,000	112 (41.2)
>75,000	160 (58.8)
Work Status	
Unemployed/retired	132 (48.5)
Full-time/part-time worker	140 (51.5)
Cancer Stage	
Stage 0 and I	216 (84.7)
Stage II and III	39 (15.3)
Surgery type	
Lumpectomy	169 (78.6)
Mastectomy	57 (26.3)
Chemotherapy	
Yes	83 (32.8)
No	170 (67.2)
Radiation therapy	
Yes	188 (74.0)
No	66 (26.0)
AET	
Aromatase inhibitor	210 (77.2)
Tamoxifen	62 (23.8)
ASK Adherence Barriers (range = 20–100)	38.2 ± 9.2
BMQ necessity score (range = 5–20)	15.8 ± 3.4
BMQ concern score (range = 5–20)	13.3 ± 3.8
IES-R Scale (range = 0–88)	20.7 ± 14.8
Breast Cancer Prevention Trial Symptom Subscales	
Vasomotor symptoms subscale score (range = 2–10)	2.7 ± 2.4
Gastrointestinal symptoms subscale score (range = 2–10)	0.6 ± 1.0
Bladder control symptoms subscale score (range = 2–10)	1.3 ± 1.8
Dyspareunia subscale score (range = 2–10)	1.9 ± 2.3
Musculoskeletal pain subscale score (range = 3–15)	4.7 ± 3.4
Weight concerns subscale score (range = 2–10)	2.6 ± 2.1
Cognitive symptoms subscale score (range = 3–15)	3.4 ± 2.9
MUSE (range = 8–32)	26.6 ± 3.8
MSPSS subscales	
Significant other subscale (range = 7–28)	23.7 ± 6.5
Family subscale (range = 7–28)	23.3 ± 6.1
Friends subscale (range = 7–28)	23.4 ± 5.8

**Table 2:** Multiple regression model of ASK20.

	β (95% CI)	*p*-Value
Race (African American vs. White)	2.47 (0.53–4.21)	0.013 *
Self-efficacy	−0.80 (−1.03–−0.58)	0.000 **
Affect	2.79 (0.61–4.97)	0.013 *
Musculoskeletal pain symptom	0.64 (0.31–0.97)	0.000 **
Weight gain symptom	0.61 (0.18–1.03)	0.006 *
Family support	−0.38 (−0.53–−0.13)	0.001 *
BMQ concern	0.64 (0.41–0.87)	0.000 **

The *p*-values were obtained through F tests during the stepwise selection method. “β” represents the beta coefficient estimate. CI: confidence interval, * *p*  <  0.05; ** *p*  <  0.001.

## Data Availability

All data generated or analyzed during this study are included in this article. Further inquiries can be directed to the corresponding author.
